# Associations between chronic conditions and oral health services utilization in older Peruvian adults: a pooled analysis of the Demographic and Health Survey 2015-2017

**DOI:** 10.4178/epih.e2020023

**Published:** 2020-04-09

**Authors:** Diego Azañedo, Diego Chambergo-Michilot, Akram Hernández-Vásquez

**Affiliations:** 1 Universidad Católica de Trujillo, Instituto de Investigación, Chimbote, Peru; 2 Escuela de Medicina Humana, Facultad de Ciencias de la Salud, Universidad Científica del Sur, Lima, Peru; 3 Universidad San Ignacio de Loyola, Vicerrectorado de Investigación, Centro de Excelencia en Investigaciones Económicas y Sociales en Salud, Lima, Peru

**Keywords:** Oral health, Mouth diseases, Aged, Noncommunicable diseases, Peru

## Abstract

**OBJECTIVES:**

This study was conducted to investigate the associations between chronic conditions (CCs) and oral health services utilization (OHSU) within the previous 6 months in older Peruvian adults (defined as those 60 years of age or more according to Peruvian law).

**METHODS:**

An analytical cross-sectional study was performed based on the 2015-2017 Peruvian Demographic and Family Health Survey. Pooled data from 13,699 older adults were analyzed. A logistic regression model was used to analyze the associations between OHSU (dependent variable) and CCs (independent variables). Tobacco consumption, obesity, educational level, age, sex, welfare quintile, area of residence, having health insurance, and natural region of residence were included as covariates in the analysis.

**RESULTS:**

The frequency of OHSU in older adults was 18.5% (95% confidence interval [CI], 17.8 to 19.3). The highest percentage point (%p) differences with regards to OHSU were found between the extreme categories of educational level (higher education vs. none or elementary school: +24.8%p) and welfare quintile (richest vs. poorest: +24.0%p). In the crude model, OHSU was associated with diabetes (odds ratio [OR], 1.46; 95% CI, 1.26 to 1.69), but this association disappeared after adjustment for covariates. Meanwhile, depression decreased the likelihood of OHSU (OR, 0.82; 95% CI, 0.72 to 0.95) in the adjusted model.

**CONCLUSIONS:**

The frequency of OHSU was low in older Peruvian adults. Regarding CCs, we found that depression independently decreased the likelihood of OHSU in the adjusted model. Our results may be useful for the development of policies aimed at achieving greater OHSU in older adults with CCs, especially in those with depression.

## INTRODUCTION

The number of older adults worldwide has increased to one billion in recent decades [1]. This trend poses major challenges for health systems due to the high levels of morbidity and mortality in this population due to chronic conditions (CCs), and this is a particularly significant problem in Latin America and the Caribbean (LAC), as the highest growth rate of older adults is occurring in that region [2].

Oral diseases are among the principal CCs in both the general and geriatric populations. The Global Burden of Disease Study reported that dental caries affected half of the world population in 2017 and was also the most prevalent condition assessed [3]. Some other prevalent oral diseases in the elderly are edentulism, dry mouth, periodontitis, and oral cancer, all of which have a major impact on general well-being [4]. Additionally, there is evidence that oral health influences self-esteem and socialization in the elderly population, which could be linked to a decrease in their quality of life [4]. Taking this into account, prevention of oral diseases through the utilization of oral health services is an important issue, and studies on the determinants of oral health services utilization (OHSU) in older adults are needed, as oral diseases are a frequent problem in this population.

There are socioeconomic and demographic barriers to OHSU. Several studies have found that inequalities in OHSU were associated with the level of education and welfare quintile [5]. Furthermore, the frequency of OHSU significantly varies with the area of residence [5,6]. These barriers are a common problem in countries with centralized health systems. In addition to inequalities in OHSU among older adults, the high burden of CCs among the elderly could further diminish the likelihood of their effective use of oral health services, mainly due to CC-related disabilities and out-of-pocket expenses related to the management of CCs, which may limit their access to health services [7,8].

The available literature supports the presence of associations between OHSU and CCs in high income countries. For instance, some studies have reported a link between metabolic conditions (e.g., diabetes) and OHSU, as in a systematic review conducted in 14 mainly developed Asian, European and American countries that found poor OHSU among people with diabetes, which could be linked to inadequate knowledge about oral health and poor oral health attitudes [9]. Furthermore, Okoro et al. [10] found an inverse association between the presence of depression and OHSU on a study conducted in the United States. Dental anxiety as a consequence of fear was proposed to modulate this association [11]. However, there is little evidence regarding the impact of CCs on OHSU in low-income and middle-income countries (LMIC).

Oral diseases pose significant challenges to health systems owing to their high economic burden and poor investments in oral health [12,13]. Thus, research into the determinants of OHSU requires studies to be conducted in LMIC, such as Peru, where the frequency of OHSU in older adults is low and several inequalities have been reported [14,15]. A better understanding of the associations between CCs and OHSU is needed to focus on early intervention and prevention as part of oral disease policies. Therefore, the aim of the present study was to investigate these associations in older Peruvian adults through a pooled secondary data analysis of the Demographic and Family Health Survey 2015-2017.

## MATERIALS AND METHODS

### Study design and data sources

An analytical cross-sectional study was performed based on the 2015-2017 Peruvian Demographic and Family Health Survey (ENDES, initials in Spanish) conducted by the National Institute of Statistics and Informatics of Peru. ENDES is a yearly population-based survey carried out using the format of the Demographic and Health Survey (DHS) program that aims to collect information about maternal health, children’s health, and non-communicable and communicable diseases. More detail about the ENDES methodology can be found in the technical reports from 2015 (https://bit.ly/2ZeFks8), 2016 (https://bit.ly/2PLFHY9), and 2017 (https://bit.ly/2PLFHY9).

The sampling for ENDES is 2-staged, probabilistic, balanced, stratified, and independently conducted in the urban and rural areas of each region. It has nationwide, regional, and urban-rural representativeness. The research units are the habitual residents of urban and rural households who spent the night before the survey in the selected household. Information is collected by direct interviews conducted by duly trained personnel, who visit the selected households to administer the survey questionnaires.

We only considered data related to older adults, defined as persons 60 years of age or more according to Peruvian Law 30490. We analyzed data from 13,699 older adults with complete data out of a total of 96,870 people aged 15 or over distributed across 107,720 households.

### Study variables

The dependent variable in this study was OHSU within the previous 6 months, which was constructed using the following questions: “Have you ever been treated in a dental service or by a dentist in your life?” and “How long ago was your last visit to a dentist?” An affirmative answer to the first question and reporting that the last dental visit was within the previous 6 months prior to the survey were considered as “yes,” while other combinations of responses were considered as “no.” Despite the lack of high-quality evidence supporting the recommendation of a 6-month interval for OHSU [16], international organizations recommend dental check-ups every 3 months to 24 months depending on individual’s needs [17]. We considered that 6 months allowed a better estimation of regular OHSU since the risk of deficient oral health increases among older adults, who present an elevated prevalence of caries, periodontal disease, and tooth loss [4].

The explanatory variables of the study were the self-reporting of diabetes mellitus (yes, no), which was evaluated with the question: “Have you ever been diagnosed with diabetes or ‘high blood sugar’ by a doctor in your life?”; the presence of high blood pressure (yes, no), defined by a participant having a mean systolic blood pressure of ≥ 140 mmHg and/or a mean diastolic blood pressure of ≥ 90 mmHg [18], or having previously been diagnosed with hypertension; the presence of at least moderate depresive symptoms, which were evaluated using the Patient Health Questionnaire (PHQ-9; yes, no), considering a PHQ-9 score ≥ 10 as “yes” [19,20]; the presence of the combinations of diabetes and hypertension (yes, no), diabetes and depression (yes, no), or hypertension and depression (yes, no); and the presence of multimorbidity (yes, no), considered as “yes” for 2 or more of the previously listed diseases.

The following covariates were included: tobacco consumption within the last 30 days (yes, no); the presence of obesity (yes, no), with a body mass index (BMI) ≥ 30 kg/m^2^ being considered as yes; educational level (none or elementary school, primary education, secondary education, and higher education); age in years, sex (male, female); welfare quintile (poorest, poor, middle, rich, richest); area of residence (urban, rural); having health insurance (yes, no); and natural region of residence (coast, highlands, jungle). The variables were selected according to the Andersen’s healthcare utilization model (with the exception of necessity variables, which are not measured in ENDES) [21] and Peruvian studies on access to health services [14,15].

### Statistical analysis

Integration, processing, and statistical analysis of the databases were performed using Stata version 14.2 (StataCorp., College Station, TX, USA). The characteristics of the sample design and the weighting factors of the ENDES were specified using the *svy* command, as well as the *subpop* option for estimating the sub-population (older adults) included in the analysis.

The variables of interest in the sample of older adults were described by simple frequencies and weighted proportions with their 95% confidence intervals (CIs). The chi-square test was utilized to assess the association between independent and dependent variables.

A logistic regression model was used to measure the associations between CCs and OHSU. Bivariate logistic regression was carried out between the variable of interest and independent variables. Independent variables showing a significant p-value and variables reported in the literature as predictors of our outcome variable were included in the multiple logistic regression, which included tobacco consumption, obesity, educational level, age, sex, welfare quintile, area of residence, having health insurance, and natural region of residence. We presented odds ratios (ORs) with 95% CIs for both models. Multicollinearity was assessed by the variance inflation factor. For all tests conducted in the study, p-value< 0.05 was considered to indicate statistical significance.

### Ethics statement

The conduct of this study did not require the approval of an ethics committee because it was an analysis of secondary data in the public domain (http://iinei.inei.gob.pe/microdatos/) that do not allow participants to be identified.

## RESULTS

**Figure 1.**
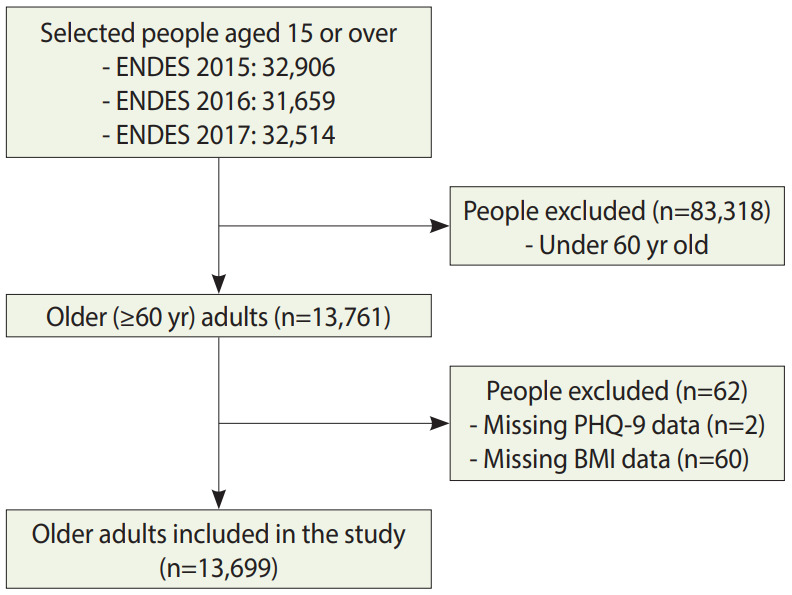
Flowchart of the selection of older adults included in the study. ENDES, Demographic and Family Health Survey; PHQ-9, Patient Health Questionnaire-9; BMI, body mass index.

The flowchart for the inclusion criteria is shown in Figure 1. In total, we included 13,699 older adults. Regarding socio-demographic characteristics (Table 1), their mean age was 70.43± 8.00 years, the most common welfare quintiles were the poorest (39.2%) and the poor (18.9%), and 82.3% had health insurance. The frequency of OHSU in the previous 6 months was 18.5% (95% CI, 17.8 to 19.3). The most frequent CCs were hypertension (47.4%) and depression (15.2%).

We found statistically significant differences in the frequency of OHSU according to the presence and absence of diabetes, depression, and obesity, as well as educational level, sex, welfare quintile, place of residence, having health insurance (yes or no), and natural region. Additionally, there was a high percentage point (%p) difference in the frequency of OHSU between the extreme categories of education (higher education vs. none or elementary school: +24.8%p) and welfare quintiles (richest vs. poorest: +24.0%p). Furthermore, a relevant percentage point difference (+10.6%p) was observed between urban-dwelling and rural-dwelling participants (Table 2).

In the crude model, OHSU was associated with diabetes (OR, 1.46; 95% CI, 1.26 to 1.69) and depression (OR, 0.63; 95% CI, 0.55 to 0.72). Regarding combinations of CCs, people with diabetes and hypertension had a higher likelihood of OHSU (OR, 1.49; 95% CI, 1.25 to 1.77) than people without this combination. In the model adjusted for smoking, obesity, educational level, age, sex, welfare quintile, area of residence, insurance, and region, the only significant finding was that people with depression had a significantly lower likelihood of OHSU (OR, 0.82; 95% CI, 0.72 to 0.95) than people without depression. Finally, no association between OHSU and multimorbidity was observed in any model (Table 3).

## DISCUSSION

In this study, the frequency of OHSU in older adults within 6 months prior to the survey was 18.5%. We found socioeconomic inequalities in OHSU, with particularly high percentage point differences between extreme categories of education and welfare quintiles. Specifically, a greater proportion of those who had higher education and those in the richest quintile utilized oral health services. In the adjusted model, the association between diabetes and OHSU became non-significant, but depression significantly decreased the likelihood of OHSU in older adults.

This study also revealed that OHSU was low in older adults. It has been reported that OHSU diminishes with age [22]. This could be attributed to the consequences of aging-related difficulties in communicating and functional dependence [23,24], which may compromise older adults’ social skills and mobility and may alter their patterns of healthcare utilization.

In addition, the Peruvian Basic Healthcare Plan does not include complex recuperative treatments, such as oral rehabilitation with dental prostheses (https://bit.ly/36YLHCE). This is an important non-covered treatment needed by older adults, since edentulism is highly prevalent in this population [4]. Moreover, these treatments are usually offered by private dental services, and this may explain why only 2 in 10 older adults who reported utilizing oral health services had their care covered by health insurance.

Several socio-demographic barriers limit OHSU in various populations. We found relevant inequalities regarding welfare quintile, education, and area of residence. This is supported by national and international literature [5,6,14,15]. A study using ENDES data reported that the annual frequencies of OHSU in older adults were low, and these percentages increased with welfare quintiles [14]. Other studies found educational and residence-based (urban vs. rural) inequalities in OHSU in adults in the United Kingdom and in older adults in Chile, respectively [5,6]. These findings are similar to ours. Favorable characteristics regarding individuals’ socioeconomic status are likely to improve their health status, since both education and welfare quintile promote knowledge of health care and access, respectively. Policies should improve the centralized health system and education on prevention to broaden the scope of OHSU [25,26].

Regarding CCs, the prevalence of OHSU in the previous 6 months was 24.3% in participants with diabetes. Although this was greater than the overall percentage of participants who received OHSU (18.5%) in our study, some studies of patients with diabetes have reported even higher prevalence rates of OHSU (between 40% and 73%) [27-29]. However, those studies evaluated dental visits during the previous 12 months, unlike our approach, which involved a 6-month interval. Other studies in older Peruvian adults have also reported low prevalence rates when evaluating OHSU over a 12-month (21.7%) or a 6-month period (24.9%) in 2014 and 2018, respectively [15,30]. Despite the pathophysiological link between oral health and diabetes [31], which involves a greater need for OHSU, in our study this association became non-significant after controlling for socioeconomic confounders. Most likely, a low knowledge of oral health, which may be determined by education, impacted the degree to which persons with diabetes were interested in OHSU. The ENDES does not report when diabetes was diagnosed; however, it would have been useful to control for this factor when analyzing this association, since a longer duration of diabetes is associated with more frequent dental manifestations. Likewise, welfare quintile—as a proxy for socioeconomic status—influences health access, and, given the fact that oral health services are not available to everyone, people in the lower welfare quintile would be likely not to receive oral health services despite having diabetes. Likewise, a similar association was described in the study of Wade et al. [32] in the United Kingdom, in which the probability of OHSU was lower in adults with 1 CC and a low educational level. Additionally, a study of 41,220 participants in Germany found that lower socioeconomic status was associated with a lower likelihood of OHSU [33]. It is important to note that some studies have reported that patients with diabetes who were better informed about oral manifestations were more likely to have good attitudes towards oral care [25]. These results show that despite the need for OHSU, education and other socioeconomic proxies have a powerful influence on the utilization of these services.

We found a high frequency of non-OHSU (86.8%) in the previous 6 months among older adults with depression. Two studies reported a lower frequency of non-OHSU (31.6-43.7%) in adults with depression in the last year [10,34]. However, aging influences depression, and it is therefore important to keep in mind that one of those studies did not include older adults, and the prevalence of older adults in the other study was 15.9%, whereas our study only assessed older adults. Moreover, OHSU was considered in the last year or 2 years in these studies, whereas we considered the last 6 months. Lengthening the study period allows more cases of OHSU to be found, with a subsequent reduction in the intensity of the association between non-OHSU and depression. The distribution of age in the study population and the method used to measure OHSU are relevant characteristics for understanding differences among studies in the frequency of OHSU in people with depression.

Furthermore, in the adjusted model, people with depression had a lower likelihood of OHSU. Several papers have suggested possible mechanisms to explain this association. For example, symptoms of geriatric depression, such as chronic pain [23,35], functional dependence [23], and fatigue, in addition to geriatric frailty, can limit the degree to which they notify others of their discomfort, leading them to avoid social situations and consequently causing reductions in their frequency of and interest in healthcare utilization. This situation may be intensified by changes in communication ability in the elderly. For example, 75% of 12,769 older adults with Medicare (United Sates) had problems with communication (listening, writing, using the phone) [33,36]. Moreover, the highest regional growth rate of older adults (in the 2015-2020 period) can be found in LAC, and since the majority of these countries are LMIC [2], they may not be able to address communication issues in this population, thereby reducing the effectiveness of healthcare services.

Another interesting explanation for the low utilization of oral health services in people with depression is their fear of visiting the dentist. People with depression may be afraid of threatening stimuli in dental situations, and this may manifest as devastating feelings of phobia and trepidation. Bernson et al. [11] reported that dental fear, anxiety, and depression were significantly higher in people with irregular dental care than in people with regular OHSU. Older adults generally cannot attend health centers on their own and must almost always be accompanied due to communication and mobility difficulties. Another study showed that the frequency of depression and anxiety was higher in patients with high dental fear than in those who had mild fear [37]. Psychological conditions are linked with negative emotions and socialization problems, which can intensify dental fear.

Due to the high frequency of OHSU in older adults with CCs, we hypothesized that multimorbidity could be associated with OHSU; however, this was not the case in our study. In contrast to our findings, Wade et al. [32] found that multimorbidity increased the likelihood of OHSU in adults in the United Kingdom. While the objective of their study was similar to ours, they included more CCs (12 vs. 4), which increased the frequency of multimorbidity in participants with OHSU (40.0% vs. 32.9%), meaning that the association could have been overestimated. Although people with multimorbidity use health services more than people without multimorbidity [38], it is erroneous to consider that all CCs have the same impact on OHSU. This is because the oral complications or manifestations of CCs vary in prevalence, extent, and severity [39,40], which may influence health-seeking behavior, and therefore, OHSU.

The results of this study document an association between depression and OHSU in older adults, showing a negative relationship between these 2 factors. Improved policies are needed to ensure a national scope of care for the minimum dental requirements of older adults, as well as policies that make this care more available for specific groups, such as people with mental health problems. New strategies must be focused on overcoming barriers, which are mainly related to education, welfare quintile, insurance and area of residence.

Regarding the limitations of this study, this was a secondary analysis of a survey; therefore, the data could have been affected by inaccuracy or memory bias. Although the investigators received previous training, there could have been errors in data collection. However, as the ENDES follows the DHS model, there is a certain methodological strength in the data collection. Consequently, the bias may have been lower than would otherwise be expected. The OHSU did not distinguish between recuperative and preventive interventions, nor were more frequent CCs such as osteoarthritis reported in older adults, which would be interesting to analyze. Furthermore, there was a 6-month window period for this survey, which could have excluded people who had utilized oral health services in the previous months. Despite these limitations, this survey is national in scope with a high level of regional representativeness, and the results are important for new national policy proposals regarding oral health in older adults.

In conclusion, the prevalence of OHSU was found to be low in older Peruvian adults. Socioeconomic variables, such as education, welfare quintile, having health insurance, and area of residence, were determinants of variation in OHSU. Regarding CCs, we found that the association between diabetes and OHSU depended on demographic and socioeconomic variables, while depression reduced the likelihood of OHSU independently of these variables. Although further research is needed, our results may be useful for the development of policies aimed at increasing OHSU in older adults with CCs, especially in those with depression.

## Figures and Tables

**Table 1.** Characteristics of the older adults included in this study, ENDES 2015-2017 (n=13,699)

Characteristics	Absolute frequency (n)	Weighted proportion (%)^1^	95% CI	
			LL	UL
Diabetes			-	
Yes	1,070	7.8	7.3	8.3
No	12,629	92.2	91.7	92.7
Hypertension				
Yes	6,499	47.4	46.5	48.4
No	7,200	52.6	51.6	53.5
Depression				
Yes	2,087	15.2	14.5	16.0
No	11,612	84.8	84.0	85.5
Tobacco consumption				
Yes	887	6.5	6.0	7.0
No	12,812	93.5	93.0	94.0
Obesity				
Yes	2,752	20.1	79.0	80.8
No	10,947	79.9	19.2	21.0
Multimorbidity				
Yes	1,681	12.3	11.7	12.9
No	12,018	87.7	87.1	88.3
Educational level				
None or elementary school	3,200	23.4	22.3	24.5
Primary	6,449	47.1	46.0	48.2
Secondary	2,382	17.4	16.6	18.2
Higher education	1,668	12.2	11.4	13.0
Age, mean±SD (yr)	70.43±8.00	-	-	
Sex				
Male	6,275	45.8	44.9	46.7
Female	7,424	54.2	53.3	55.1
Welfare quintile				
Poorest	5,376	39.2	37.3	41.3
Poor	2,594	18.9	18.0	19.9
Middle	1,994	14.6	13.7	15.4
Rich	1,903	13.9	13.1	14.8
Richest	1,832	13.4	12.4	14.5
Area of residence				
Urban	7,683	56.1	53.8	58.4
Rural	6,016	43.9	41.6	46.2
Health insurance				
Yes	11,281	82.3	81.5	83.1
No	2,418	17.7	16.9	18.5
Natural region of residence				
Coast	5,161	37.7	35.6	39.8
Highlands	5,976	43.6	41.4	45.9
Jungle	2,562	18.7	17.2	20.3

ENDES, Demographic and Family Health Survey; CI, confidence interval; SD, standard deviation; LL, lower limit; UL, upper limit.

1 The weighting factor and sample specifications of the ENDES were included.

**Table 2.** Oral health services utilization within the previous 6 months according to the characteristics of older adults^1^

Characteristics	Oral health services utilization within the previous 6 mo, % (95% CI)		p-value^2^
	Yes (n=2,540)	No (n=11,159)	
Total	18.5 (17.8, 19.3)	81.5 (80.7, 82.2)	-
Diabetes			<0.001
Yes	24.3 (21.8, 27.0)	75.7 (73.0, 78.2)	
No	18.1 (17.3, 18.8)	81.9 (81.2, 82.7)	
Hypertension			0.201
Yes	19.0 (18.0, 20.0)	81.0 (80.0, 82.0)	
No	18.1 (17.2, 19.1)	81.9 (80.9, 82.8)	
Depression			<0.001
Yes	13.2 (11.8, 14.7)	86.8 (85.3, 88.2)	
No	19.5 (18.7, 20.3)	80.5 (79.7, 81.3)	
Tobacco consumption			0.756
Yes	18.2 (15.7, 20.8)	81.8 (79.2, 84.3)	
No	18.6 (17.8, 19.4)	81.4 (80.6, 82.2)	
Obesity			<0.001
Yes	24.2 (22.6, 25.9)	75.8 (74.1, 77.4)	
No	17.1 (16.3, 17.9)	82.9 (82.1, 83.7)	
Multimorbidity			0.615
Yes	32.9 (30.5, 35.3)	67.1 (64.7, 69.5)	
No	32.3 (31.2, 33.3)	67.1 (64.7, 69.5)	
Educational level			<0.001
None or elementary school	11.3 (10.2, 12.4)	88.7 (87.6, 89.8)	
Primary education	15.3 (14.4, 16.3)	84.7 (83.7, 85.6)	
Secondary education	24.7 (23.0, 26.5)	75.3 (73.5, 77.0)	
Higher education	36.1 (33.7, 38.5)	63.9 (61.5, 66.3)	
Age, mean (95% CI)	69.2 (68.8, 69.5)	70,8 (70.6, 70.9)	<0.001^3^
Sex			<0.001
Male	19.7 (18.7, 20.9)	80.3 (79.1, 81.3)	
Female	17.5 (16.8, 18.5)	82.5 (81.5, 83.4)	
Welfare quintile			<0.001
Poorest	11.4 (10.5, 12.4)	88.6 (87.6, 89.5)	
Poor	15.6 (14.2, 17.2)	84.4 (82.8, 85.8)	
Middle	20.1 (18.4, 21.9)	79.9 (78.1, 81.6)	
Rich	24.8 (22.9, 26.7)	75.2 (73.3, 77.1)	
Richest	35.4 (33.1, 37.7)	64.6 (62.3, 66.9)	
Area of residence			<0.001
Urban	23.2 (22.2, 24.2)	76.8 (75.8, 77.8)	
Rural	12.6 (11.7, 13.7)	87.4 (86.3, 88.3)	
Health insurance			<0.001
Yes	19.9 (19.0, 20.7)	80.1 (79.3, 81.0)	
No	12.3 (11.0, 13.7)	87.7 (86.3, 89.0)	
Natural region of residence			<0.001
Coast	23.0 (21.7, 24.2)	77.0 (75.8, 78.3)	
Highlands	16.5 (15.4, 17.7)	83.5 (82.3, 84.6)	
Jungle	14.3 (12.9, 15.8)	85.7 (84.2, 87.1)	

Values are displayed as weighted % of the row unless otherwise indicated. CI, confidence interval.

1 The weighting factor and sample specifications of the Demographic and Family Health Survey were included.

2 Chi-square test.

3 t-test.

**Table 3.** Association between oral health services utilization within the previous 6 months and chronic conditions

Variables	cOR (95% CI)	p-value	aOR (95% CI)^1^	p-value
Diabetes				
No	1.00 (reference)		1.00 (reference)	
Yes	1.46 (1.26, 1.69)	<0.001	1.07 (0.92, 1.24)	0.401
Hypertension				
No	1.00 (reference)		NA	
Yes	1.06 (0.97, 1.15)	0.293	NA	-
Depression				
No	1.00 (reference)		1.00 (reference)	
Yes	0.63 (0.55, 0.72)	<0.001	0.82 (0.72, 0.95)	0.008
Diabetes and hypertension				
No	1.00 (reference)		1.00 (reference)	
Yes	1.49 (1.25, 1.77)	<0.001	1.08 (0.89, 1.30)	0.433
Diabetes and depression				
No	1.00 (reference)		NA	
Yes	1.23 (0.84, 1.80)	0.293	NA	-
Hypertension and depression				
No	1.00 (reference)		1.00 (reference)	
Yes	0.67 (0.55, 0.81)	<0.001	0.83 (0.68, 1.01)	0.057
Multimorbidity				
No	1.00 (reference)		NA	
Yes	0.95 (0.83, 1.09)	0.485	NA	-

All estimations included the Demographic and Family Health Survey weighting factor and sample specifications. cOR, crude odds ratio; CI, confidence interval; aOR, adjusted odds ratio; NA, not applicable.

1 Adjusted for tobacco consumption, obesity, educational level, age, sex, welfare quintile, area of residence, having health insurance, and natural region of residence.
